# The Interplay between Social and Ecological Determinants of Mental Health for Children and Youth in the Climate Crisis

**DOI:** 10.3390/ijerph18094573

**Published:** 2021-04-26

**Authors:** Maya K. Gislason, Angel M. Kennedy, Stephanie M. Witham

**Affiliations:** Faculty of Health Sciences, Simon Fraser University, 8888 University Drive, Burnaby, BC V5A 1S6, Canada; angel_kennedy@sfu.ca (A.M.K.); stephanie_witham@sfu.ca (S.M.W.)

**Keywords:** social determinants of health, ecological determinants of health, mental health, psychosocial, population health, health equity, eco-social health, children, youth

## Abstract

Children and youth are showing increasing levels of mental health distress due to the climate crisis, characterized by feelings of sadness, guilt, changes in sleep and appetite, difficulty concentrating, solastalgia, and disconnection from land. To gain a deeper understanding of the relationship between climate change and children and youth’s mental health, we conducted a rapid review and a thematic analysis of the results in NVivo 12. Our findings show that children and youth experience a plethora of direct and indirect effects from climate change and this impacts their mental wellbeing in diverse and complex ways. Young people also have varied perceptions of climate change based on their social locations and many are dealing with feelings of immense worry and eco-anxiety. The mental health impacts of climate change on children/youth are tied to Social Determinants of Health (SDoH) but also need to be understood in relation to the Ecological Determinants of Health (EDoH). Through an eco-social lens, this paper explores these conceptual issues and uses them to provide a framework for understanding the interplay of social and ecological determinants of mental health for children/youth.

## 1. Introduction

In the current historical moment, a contradiction is arising where generally people are living longer and healthier lives; however, for the youngest generations, the assumptions of unending economic growth, social stability, and increasing health and wellbeing across the life-course are not experienced ubiquitously. At an early age, children (defined by the Canadian Census as individuals between the ages of 0–14) and youth (defined as individuals between the ages of 15–24) who have always lived under the specter of climate change are facing a barrage of information which suggests that the planet as we know it is already, and will increasingly, change for the worst. A significant, and growing impact, on children and youth’s mental health, therefore, is climate change. Globally, children/youth are exhibiting deepening distress around climate change, and will be disproportionately burdened by it across their life-course [1]. Whether due to direct impacts from climate events or as a result of growing up in a discursive space infused with climate anxiety, children/youth in Canada and around the world are showing increasing levels of mental health distress from climate change [2]. Furthermore, it is predicted that without accelerated intervention, climate change will impact the health of people at every stage of their lives and in both short- (e.g., health care visits) and long-term (e.g., lost potential) ways [3]. These concerns are further exacerbated given that children/youth are developing both psychologically and physically and more susceptible to environmental elements than adults [4], in part due to the time they spend playing outside in close contact with nature [3]. Additionally, their physiological and mental development can slow down or be halted by the unpredictable consequences of increased climate impacts [3]. It is posited that impacts from climate change events start before birth when birthing bodies are exposed to environmental stressors that impact the baby in utero [4]. As a result, this phenomenon raises significant questions in relation to childhood development and precipitates a new set of considerations that must be attended to when considering the causes and consequences of mental health disorders on society.

Through an approach informed by intergenerational climate justice, this paper reflects the view that the current generation has a responsibility to protect the climate system for the benefit of present and future generations of children and youth [4]. To date, research on climate change and health has tended to be organized around climate events, such as flooding, sea level rise, or forest fires, mental and physical health harms, and individual pathologies [3]. Additionally, while the physical health impacts of climate change are well researched [5], and although there is increasing agreement around the mental health impacts of climate change, knowledge gaps exist about the impacts to specific populations of children/youth. This paper argues that an important approach to take when working at the interface of children/youth’s experiences of climate change and mental health is to consider the interplay between the Social and Ecological Determinants of Health.

While the Social Determinants of Health (SDoH) are increasingly well understood as socio-economic, cultural, and environmental conditions that affect health outcomes, lesser known are the Ecological Determinants of Health (EDoH). These are distinct from the field of environmental health and extend beyond the limits of the ‘environmental’ determinants to the earth system. Within the context of ecosystem services, fundamental needs like water, food, and natural resources are impacted by global environmental changes like biodiversity loss, pollution, resource depletion and climate change. An eco-social approach grasps the holistic nature of public health as it impacts the most basic necessities for health and shows that they are underrecognized at all levels of government [6]. For topics that arise in the interplay between the social and natural world, the EDoH offer an important complement to the SDoH as together they form crucial components to environmental and social health and wellbeing [7]. An ‘eco-social’ approach centers the idea that social and ecological factors are key components of the health and wellbeing of current and future populations. While environmental and ecological approaches are complementary, the EDoH strengthens attention to how ecosystems and ecologies are intrinsically linked to both human and animal health. Examining the determinants of children/youth’s mental health illuminates the limits to knowledge that would occur if genetic, biological and family factors, or a focus on individual’s exposures to emotional or physical adversity or abusive conditions alone were used to study contemporary socio-environmental issues and their impacts on children/youth’s mental health [8].

Foregrounding an equity lens is also critical when studying the eco-social impacts of climate change on children/youth’s health. People and communities differ in their exposures, inherent sensitivity, and adaptive capacity to respond to and cope with climate events [9,10,11]. Presently, the literature suggests that while new forms of climate-influenced mental health impacts are emerging, such as ‘eco-anxiety’ and ‘solastalgia’, climate change predominantly aggravates existing risk factors and conditions, disproportionately impacts vulnerable populations, and further taxes already strained mental health services [12]. These psychological impacts worsen overall health, diminish productivity, and reduce quality of life.

Although we know that climate change intensifies and complexifies vulnerability in the lives of children and youth generally, the impacts to specific populations and conditions are not yet well understood. A ‘social inequities in health’ approach offers a way to understand how processes and relationships of structural inequities and institutionalized forms of exclusion and marginalization can lead to climate change disproportionately impacting a range of equity-deserving groups of children and youth. Necessarily, as the evidence for this complex interplay of social, environmental, and health factors cannot be studied easily within a laboratory setting, researchers must bring together novel mixed-methods approaches, particularly in contexts where equity-informed data are missing, and the living experiences of equity-deserving groups have been underreported. Culturally sensitive data are of key importance, as they highlight differences in experience and impact—in particular in the Canadian context, for Indigenous peoples.

Ultimately, the goal of this paper is to identify how diverse social experiences of ecological factors may contribute to process which result in the development of mental disorders and mental health challenges in children and youth, as well as to better understand how they can and do recover from them. This paper is intended to offer novel insights into how to work at a population scale to enhance research and policy development at the nexus of mental health and climate change research.

## 2. Methods

### 2.1. Search Strategy

A rapid review was conducted as it can produce similar validity to a systematic review yet is done within a shortened time period [13]. The objective of the review was to conduct literature searches to find relevant studies focusing on the impact of climate change on children and youth’s mental health.

Three key questions guided this rapid review:

1. What are the direct and indirect impacts of climate change on children/youth’s mental health in Canada?
2. What are children/youth’s perceptions or views of climate change and how do they impact their mental health and wellbeing?
3. In what ways can taking action on climate change through community projects strengthen and build resilience in children/youth in the age of climate change?

After considering our research questions, sources were gathered from both academic and non-academic databases by co-authors A.M.K. and S.M.W. to capture the discourse around climate change, mental health and children/youth. Studies from other interrelated literature reviews were also used to inform the paper, including grey literature, as this is where information around climate events is often first reported. This strategy reduced our study bias towards only peer reviewed literature. For instance, Health Canada, the Canadian Institute for Health Information, Canadian Association of Research Libraries (CARL), OpenDOAR, and the Assembly of First Nations were reviewed.

### 2.2. Review Methods

**Figure 1:**
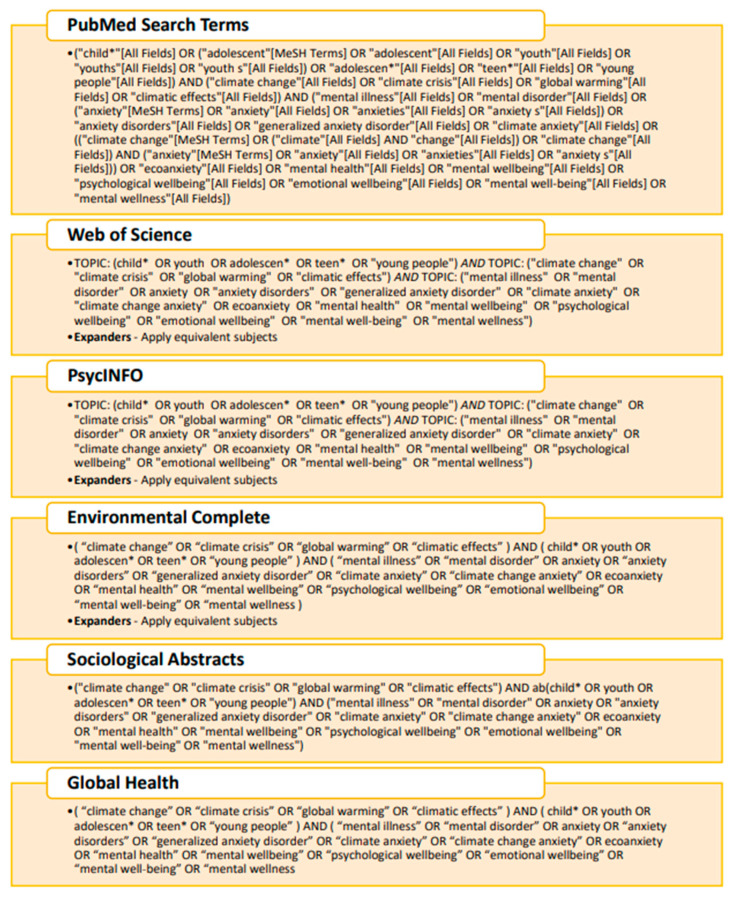
Keyword search strategy.

Before initiating the search process, coauthors A.M.K. and S.M.W. consulted with a librarian at Simon Fraser University to gather input on search strategy and keywords. Sources were collected from PubMed, Web of Science, PsycINFO, Environmental Complete, Sociological Abstracts, and Global Health databases, using relevant search terms (see Figure 1 for keyword searches). Although this number of databases would give multiple duplicates, it improved rigor by ensuring researchers found more articles on the topic.

**Figure 2:**
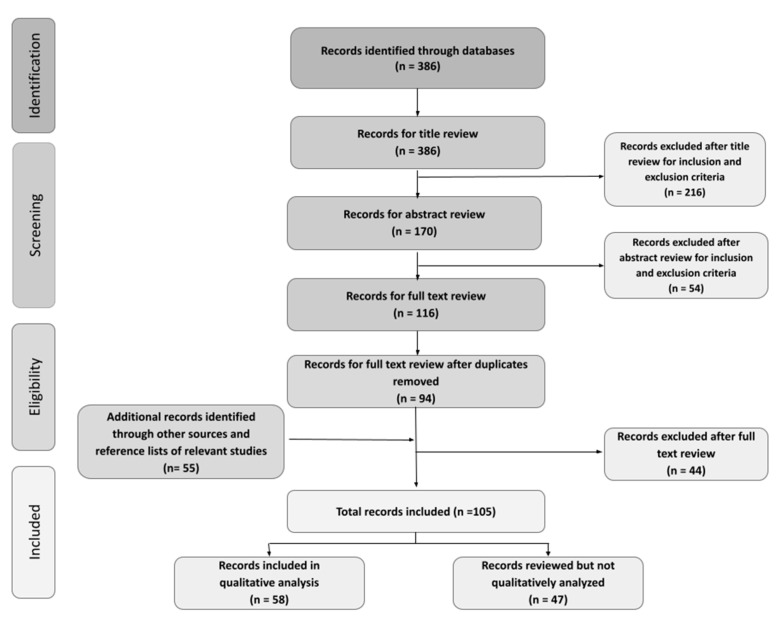
PRISMA diagram of search strategy and analysis.

We then developed inclusion and exclusion criteria that was used to screen for titles and abstracts. We included studies that were both peer-reviewed and grey literature from the past 10 years (November 2010–2020) that were published in OECD countries (Canada, Australia, US, UK, Netherlands, Sweden, Portugal, and Finland) as our intention was to include any relevant studies and not limit to Canada. Articles could have a global focus so long as the topic related to Canada as well. Records needed to explicitly address at least two of the following themes in the abstract or title: climate change, youth/children, and mental health. Inclusion criteria were left relatively open to include relevant studies on a novel topic; however, this could be considered a limitation of our study. For this specific review, papers were not included if they were non-English or had a COVID-19 or biomedical focus, unless it explicitly considered an Ecological Determinants of Health. Furthermore, articles were examined to consider inclusion of Equity, Diversity and Inclusion and Gender-Based Analysis Plus relevant terms. A total of 386 records were identified through database searches, and 170 titles were identified for abstract screening. The abstracts of 116 articles were deemed relevant and 58 articles were retained for our analysis, as identified in Figure 2. Duplicates were removed after abstract review instead of in the title screening phase so that both researchers could ensure that relevant articles were not removed at an earlier phase by another team member. This allowed the researchers to develop a high degree of agreeability in finding the same articles relevant. Both researchers worked independently for the initial screening to reduce bias and mistakes, justifying the relevance of the studies included. In situations where researchers disagreed on article inclusion, the PI (MKG) heard their views and made the final decision.

### 2.3. Analysis Methods

Following inclusion and exclusion criteria review, a multi-stage thematic analysis [14] of the 58 articles was undertaken in NVivo 12 (QSR International, Chadstone, Victoria, Australia) to capture important themes, terms, and quotes from the selected studies. Thematic analysis is an analysis methodology where researchers search for emerging themes in the data which aid in describing the phenomenon of focus and which become the categories of analysis [15]. This process further involves reading through the data, identifying key themes, revisiting the data, and refining themes as the researchers engage in coding. As such, first, the articles were read by co-authors A.M.K. and S.M.W. so they could develop familiarity with the data, reflect on initial trends and interpretations of the articles, and create an interim codebook which was then revised throughout the analysis and inductively compiled. The codes included climate change impacts on mental health for youth and children, their perceptions of climate change, importance of resilience building, proposed interventions, methodologies used, and related calls to action.

## 3. Conceptual Frameworks

The literature review and analysis conducted by our team was grounded in a series of conceptual frameworks which guided our approach to capturing the inherent complexity when working at the intersection of climate change, children/youth, and mental health, as well as aiding in our approach to evaluating results and reflecting on future steps.

The impacts of climate change on youth and children’s mental health underscore the need to develop a new arena of public health research and practice in mental health which works upstream to promote the resilience, health, and wellbeing of children/youth. A ‘whole person, whole community, whole of life, and whole of planet’ [16] approach—which seeks to promote the health and wellbeing of people in the context of their own lives and communities—was used. This approach also reflects Indigenous insights into wellness which underscore the fundamental importance of relationships with self, family, community, nature, and spirit [17]. Reflecting research and policy formation mandates in the Canadian context, our research was also informed by the principles of Equity, Diversity and Inclusion (EDI), Gender Based Analysis Plus (GBA+) and Cultural Safety. As a result, intersectionality theory was used to ensure that this research considers the realities of a diversity of children/youth and to conduct an analysis that acknowledges their heterogeneous social identities, social locations, and social experiences [18]. These approaches are valued as they deepened our scholarship on the intergenerational justice dimensions of the interplay between Anthropogenically driven climate change, children and youth, and their mental health and wellbeing.

A complementary conceptual framework we utilized in this research is informed by the Framework for Collaborative Action on Health and Climate Change [19], developed by the SHIFT Collaborative, which includes:

1. Community engagement;
2. Multi-sectoral collaboration;
3. Political engagement;
4. Healthy public policy;
5. Asset-based community development.

This framework helps to connect desk research to knowledge mobilization initiatives as it calls for explicit attention to developing evidence that can be meaningfully shared with communities and multi-disciplinary professionals when engaging in intersectoral collaboration [20]. When conducting eco-social and equity informed research, it becomes clear that in order to advance work which connects climate change adaptation and mitigation, community resilience building and the provision of supports and interventions for the mental health of children and youth, collaborations between a range of new and unusual allies is required. Asset-based Community Development (ABCD) approaches offer one way to engage in this work as it focuses on identifying and leveraging community priorities and assets, conducting research, generating new knowledge, and repeating the process while engaging with the community and building relationships throughout.

**Figure 3:**
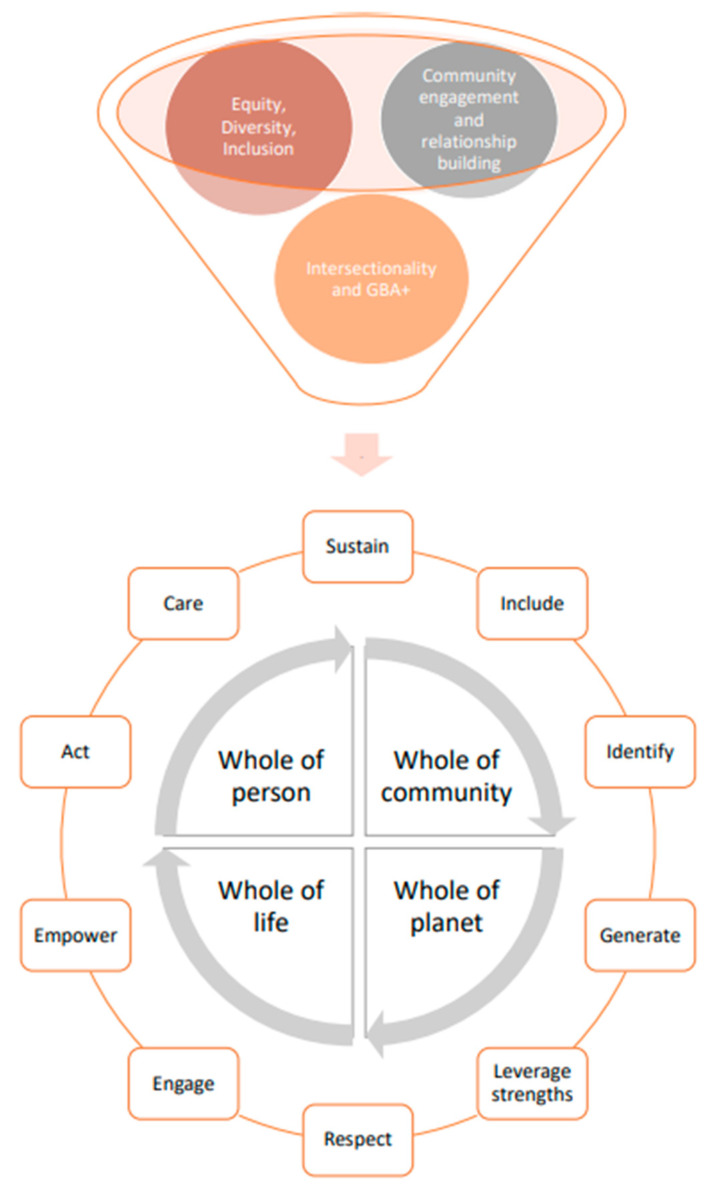
Guiding conceptual framework.

A widely shared insight emerges about the importance of co-benefits thinking as a way to guide work across a range of scales and sectors to develop and sustain innovative and locally-relevant community and environmental health promoting activities [21]. When engaging in research with children/youth and by children/youth, the Centre of Excellence for Youth Engagement’s (CEYE) encourages researchers to consider engagement across the levels of the Individual, Social, and Systems to promote and sustain meaningful involvement [22]. Often referred to as the ‘head, heart and feet of engagement’, the intention is for research and practice to include cognitive (learning new things), affective (pleasure from participating), behavioral (active engagement) and spiritual components [22]. In sum, our work was informed by the integration of these frameworks to enrich research on the complex interaction of climate change, mental health, and children/youth (see Figure 3).

This figure represents the principles of equity, diversity, inclusion, community engagement, relationship building, intersectionality, and GBA+ that ground our work. Key actions for future work include empowering equity-deserving populations, identifying gaps in mental health provisions, generating community-centered knowledge; leveraging community strengths; centering respect, health equity, and climate justice; and engaging meaningfully with community in intersectoral ways.

## 4. Results

By conducting a rapid review informed by the methodology and conceptual lens as described above, this literature review offers an equity-centered investigation into key themes around how youth and children are experiencing, thinking about, and engaging with climate change, as are discussed below (please see Table S1).

### 4.1. Direct and Indirect Effects of Climate Change on Children and Youth

In order to produce research which builds an evidence-based understanding of children and youth’s mental health in the age of Anthropocene-driven driven climate change, it is important to understand how children/youth are being affected generally. It is recognized that the climate crisis is one of the greatest human rights challenges of our time and spans the right to life, health, food, water, housing security, and the rights of Indigenous peoples. Climate change also compounds and magnifies existing inequities and does so in both direct and indirect ways as climate events vary across temporal and spatial scales and range in their severity.

The gradual nature of environmental degradation can cause chronic community, familial, and individual stress from displacement as well as loss of access to resources, connection to land and place [23]. Evidence suggests that young people are experiencing ecological grief—defined as the loss of nature and ecological surroundings [24]—which can lead some to feel angry, frustrated, and helpless [25]. Indigenous youth can experience unique cultural, community, geographical and social impacts from climate change especially in the arenas of the interconnectedness of land, community, and individual wellbeing [26,27]. For example, adverse environmental changes directly affect health and nutrition, sense of identity, community cohesion, and traditional learning for youth in northern Indigenous communities [26].

While the literature generally focuses on the direct impacts of climate change on physical health, the indirect effects to mental health largely come from lived experiences of extreme weather events and disaster situations when children and youth experience trauma and other related health disorders [23,28,29]. Indirect impacts of climate change events also includes climate change exacerbating socioeconomic inequalities and broader social determinants of health during and in the aftermath of disaster events [23,29].

### 4.2. Children and Youth’s Perceptions of Climate Change

Understanding perceptions about climate change for children and youth is tied to their general awareness, beliefs, and concerns about climate change. For instance, a national survey from the Australian Childhood Foundation has considered indicators such as feeling troubled by the state of the world and thoughts around the seriousness of climate change and its impacts [30,31]. Other studies have acknowledged climate change effects in respect to a sense of climate related stress or worry, or a loss of security for the future [28]. However, there is evidence that youth do not experience the same level of concern about climate change as children, and some studies have found that although youth associate health problems with climate change, they see the issue as a future problem that does not impact their lives now [32]. These perceptions can come from lived experiences as well as narratives projected by the media, education, family as well as from personal connections with nature [28,30].

The impacts of climate change are occurring against a backdrop where children and youth are currently experiencing considerable mental health issues for their age group, and are “feeling less in control of their lives than at any point in recent history” [33]. The literature shows that youth are often aware of the impact climate change has on their lives and the planet, and that they use a variety of mechanisms (adaptive and maladaptive) to cope with these realities. Children and youth below the voting age may also feel frustration with their perceived inability to participate democratically [34]. In addition, children and youth’s perceptions of climate change-related risk varies across several markers of privilege and social locations [35].

Perceptions of the impacts of climate change are also significantly consequential. Research shows that most young people around the world know about climate change, many express worry about its impact on their lives, and several believe that the world will end during their lifetime as a result [36]. Furthermore, a majority of youth ages 10- to 12-years old experience feelings of fear, helplessness, worry, sadness, anger, and anxiety due to climate change [30,33,34,36]. Moreover, bleak narratives of climate change in the media and global climate strikes remind young people of the issues that they currently face and which will follow them into the future [32]. The influence from peers and the media makes it difficult for parents to control the messages their children hear [37]. Negative publicity on environmental issues is ubiquitous and evidence suggests that children and youth may not be equipped to handle the influx of information [30]. However, the recent global ‘school strikes’ for climate change led by activist Greta Thunberg and allies, has also given young people the perception that organized activism is a way to show they have a voice and to hold decision makers accountable [30,38].

### 4.3. Community Action on Climate Change and Resilience

Resilience building in youth is important as it allows them to be active agents and protagonists for change [37]. Focusing on the negative impacts of climate change is a ‘narrow framework’ and can limit discourse about, and possibility for, resilient and creative approaches to solutions [39,40]. Similarly, focusing on only individualistic approaches weakens the importance of community and educator’s roles in supporting young people [39,40]. In contrast, strength-based community and participatory approaches shift away from deficit-based concepts and provide a stronger mental health ecosystem to reduce the burden for future generations [23,36]. The importance of supporting the mental health resilience of youth and children is further emphasized during the COVID-19 pandemic. Children and youth are now facing new experiences of disempowerment with the various lifestyle changes which have accompanied the pandemic. Furthermore, the mental health of youth/children is deteriorating, further validating the importance of not only mitigating the impacts of climate change, but also supporting the mental health resiliency of youth and children.

**Figure 4:**
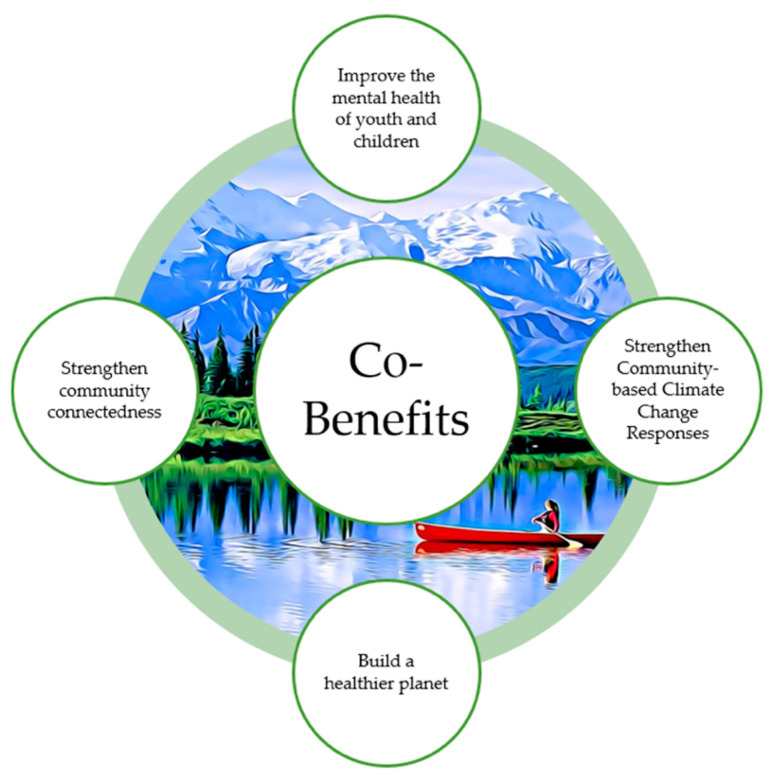
Co-benefits of working at the climate change, mental health, children/youth intersection.

Strengths- and asset-based interventions and frameworks can provide additional co-benefits to climate mitigation and preparedness, as represented in Figure 4. Strengthening community and social networks can play a powerful role in local responses and co-operation building [41]. Research shows that youth can become more empowered through local activism, self-expression, and can contribute novel and creative responses to climate change [25]. In addition, young people have an opportunity to learn practical skills through climate initiatives and engagement with nature which can also foster positive adaptive coping behaviors [36]. Impressively, the global climate strikes have shown young people’s ability to organize quickly and effectively as engaged citizens in a world they are growing up into and will eventually govern [40].

**Figure 5:**
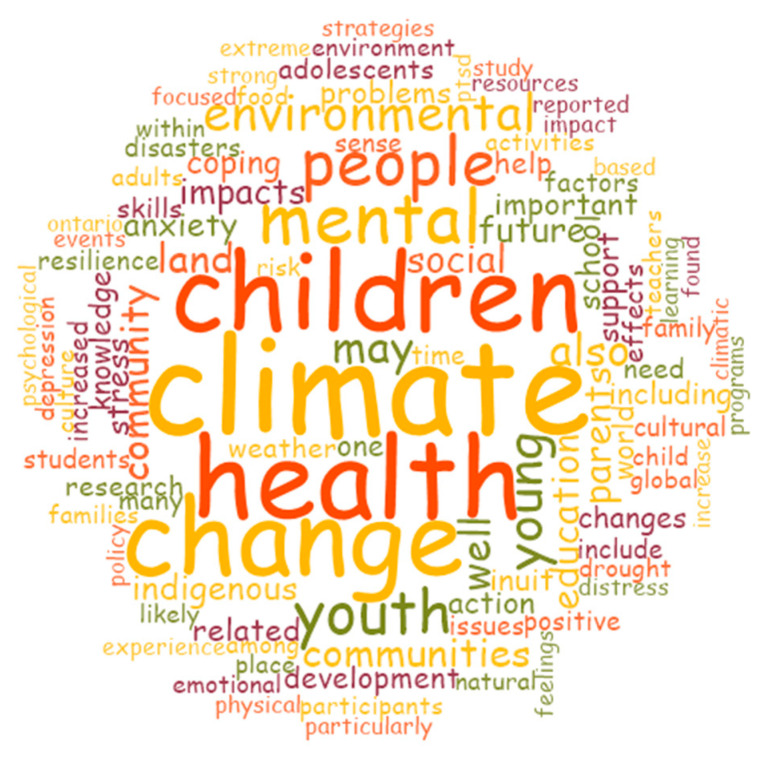
Word cloud of most frequent words in the articles included in the analysis.

Keywords pulled from our literature review results (See Figure 5) show that themes of depression, distress, and drought appear at the intersection of children/youth, climate change, and mental health. However, it is also evident that strengths-based insights are found when mitigating health inequities for children and youth growing up in a time of climate uncertainty, including themes around ‘strategies’, ‘strength’, ‘coping’, ‘skills’, ‘resilience’, ‘future’, ‘culture’, ‘positive’, and ‘change’.

Since children and youth—the future generation of leaders—are impacted by climate effects, a shared responsibility from older adults and younger generations is needed to shape policy for climate strategies [32]. Since the mental health of children/youth can have an impact on their flourishing across their life-course, this emphasizes the importance of exploring the acute effects and anticipated impacts of climate change on children and youth’s mental health now. Our findings indicate that in Canada, youth gain a sense of control, governance, representation, and autonomy when their voices are centered in community climate action approaches, as is evident in Hart and colleagues’ words: taking “action through playing a meaningful role in the face of adversity can offer a kind of psychological protection by helping children/youth to feel more in control, more hopeful, and more resilient” [42] (p. 93). Additionally, integrating connection to land, water, and animals in climate change mitigation centers a decolonizing eco-social gaze towards more equitable approaches to achieving intergenerational justice.

## 5. Discussion

When looking through the existing literature and considering issues of experiences, perceptions, responses, and actions to climate change, we begin to understand the complexity that exists in the lives of children and youth who experience both direct and indirect effects of climate change. The literature emphasizes how the interplay between social, ecological, and psychological interactions have the potential to manifest health harms for children and youth. This intersection also provides opportunities to promote the resilience of current and future generations of children and youth.

This research highlights theoretical insights and conceptual frameworks that can be used by researchers, educators, practitioners, parents, and policy makers who are interested in learning how to integrate considerations of youth and children’s mental health into climate change responses. Further opportunities exist for including children and youth’s perspectives and local knowledge systems into climate action and for imagining future roles for youth and children as change-makers in creating sustainable climate policy and community development initiatives.

Our findings underscore the importance of cultivating work that looks at the climate change–mental health nexus using group-level initiatives which have the co-benefit of strengthening community-based responses to climate change and improving mental wellbeing [21]. Consolidating this knowledge can assist in the identification of strategies that can effectively help to reduce mental health risks, offer upstream attention to the causes of harm, and build resilience in children and youth through integrating them into existing community networks, services and programs [12]. Therefore, it is crucial to initiate novel research strategies and strengthen existing intersectoral collaborations between the health sector and those jurisdictions responsible for climate education, adaptation, and mitigation. Although interventions and research may be site specific, the knowledge collected in the process of this work can be fed directly back into improving the health and wellbeing of children and youth across Canada, and around the world.

These findings can also be applied to other country contexts given the ubiquitous nature of the climate crisis. While common climate crises may differ based on region (e.g., forest fires, flooding, droughts, landslides, tsunamis, sea-level rise, ice melt, etc.) [5], the occurrence of climate change-related mental health stressors on children and youth seems consistent. This speaks to the importance of global awareness of health professionals to the impact that climate events can have on children and youth [43]. The global climate action school strikes reveal that children and youth are concerned about the climate crisis and are calling for change all around the world [39]. Additional importance is that of social, political, and economic contexts which vary by region. These various factors contribute to the impact that climate change has on economic and housing stability, food sources, education obtainment, violent events, social interaction, connection to culture, disease prevalence, and displacement, among others [41,44,45]. Despite these contextual differences, using an intersectional lens to acknowledge the role of climate change in the mental health and subsequent life course functioning of children and youth is paramount.

While literature reviews play an important role in building foundational knowledge, these reviews also allude to the need to take this knowledge further and foster a deeper understanding around children and youths’ responses to climate change. This research points to the importance of continuing to identify and consolidate the available evidence on the direct, indirect, and mediated impacts of climate change on children and youth’s mental and physical health, and on what protective factors and health promoting interventions can prevent and mitigate against these impacts [46]. Additionally, it is important to cultivate work that looks at addressing local climate issues in the context of cities, rural, remote, northern, island, and traditional territory community contexts; conducting research and community-based climate actions not only for children and youth, but with them. It also calls for integrating findings into future policies and practices to build capacity in the mental healthcare sector in Canada by integrating mental health support into existing healthcare facilities and programs. This is particularly important in rural and remote communities with limited mental health programming and funding. Other questions which are important to answer include: 1. How can local actions on climate change promote and protect children and youth’s health and wellbeing? and 2. What factors make these actions attributable to improvements in mental health and wellbeing?

The two questions stated above are distinct but linked and offer a stepwise approach for understanding the impact of engagement, states of mental health and wellness, and factors that link the two in ways that produce positive outcomes for children/youth who participate in eco-social health research. Of additional importance is the practice of researchers engaging with and supporting those who work with youth and children. In particular, those using age-appropriate ways to empower children/youth to lead interventions, identify and work on local responses to climate change, and address issues of climate change and its relation to their personal experiences, health and wellbeing. Children and youth do best when the information they are taught and the challenges they are asked to address are presented in age and stage appropriate ways. Using ‘hearts, hands and heads’ requires that projects be local and tangible in order for children/youth to be able to observe a problem and identify the factors that could be manipulated to affect positive change. It is also important that their change-making efforts produce a positive and measurable outcome that engenders a sense of achievement. The older children and youth become, the more complexity they can manage, and therefore the tasks and goals they may decide to tackle can have both a local element and a larger system dimension [1]. However, even teenagers, particularly in the context of climate change, benefit developmentally from the experience of making a positive difference [1]. Further steps can also be developed, such as cultivating new conversations, inspiring others to act, teaching friends and family members about new strategies, and/or connecting projects with each other to build capacity or scale up an initiative. Projects particularly suited to children/youth include arts-based activities [47], gardening, nature-based projects [48], or solving a discrete planning problem in a local environment, such as in relation to an issue at a school or in a neighborhood [37].

The Gold Standard for working with children/youth to manage stress, distress, trauma and anxiety is Cognitive Behavioral Therapy (CBT) [46]. When working in a space of focusing on mental health and wellness rather than mental illness diagnosis and treatment, CBT can be used for the key insights it brings to developing adaptive ways of managing challenging life situations. More specifically, CBT encourages the development of coping mechanisms amidst stressors such as direct and indirect mental health impacts of climate change. CBT typically includes four steps:

1. Identify troubling situations or conditions in your life;
2. Become aware of thoughts, emotions and beliefs about these problems;
3. Identify negative or inaccurate thinking by recognizing patterns of thinking and behavior that may be contributing to the problem;
4. Reshape negative or inaccurate thinking.

The goal is to empower children and youth with the tools that enable them to cope with challenging situations in health promoting ways, thereby promoting resilience. Other tools of importance when working at the intersection of climate change and mental health includes a free and gold standard tool used by child psychologists, The Screen for Child Anxiety and Related Disorders (SCARED) measurement instrument [49]. This tool can be utilized, and elaborated upon by integrating relevant questions from the Child Health BC’s updated Youth Health and Well-being Indicators (scheduled for release in 2021) [50], the validated Environmental Distress Scale [51], the Canadian Index for Child Health and Wellbeing [52] and the Mental Health Indicators for Canada [53]. In taking an intersectionality and GBA+ approach, other important tools and processes include self-reporting of perceptions of health and engagement in maladaptive behaviors (as determined by the individual), as well as qualitative interviews. These can be conducted with children/youth, family members and allied public health promoters on the perceived impacts to children and youth’s mental health and reflections on the mechanisms of these attributions. Through individual and focus group interviews, rich descriptions can be triangulated with participant observations to fill gaps that other tools cannot fill. These key areas are crucial to understand in order to stave off despair, improve the health and wellbeing of children and youth, elevate their voices, and build and sustain healthy communities and a healthier future.

This work calls for Integrated Knowledge to Action (iKTA) [54] which is necessary to foster partnerships with a range of experts, social organizations, intergenerational collaborators, mentors, and community organizations to develop a series of projects with and for children/youth from diverse communities, geographies, and socio-cultural backgrounds. Community-based projects and insights are central in developing actions on climate change at the local level. IKTA can provide a framework for building collaborations between children/youth and intergenerational allies using a variety of tools and processes including art, education, and techniques in climate adaptation and mitigation. Outputs from interdisciplinary research should be diverse due to the diverse social locations and backgrounds of the participants involved. Outputs may include academic research papers, conference presentations and policy briefs that are co-produced with children/youth, and/or knowledge translation materials actively defined by children- and youth-collaborators with a goal of feeding knowledge into the communities, groups, and institutions that are important in their lives [55,56,57]. Knowledge Synthesis Reports and briefs can be effective tools when advocating for system-level change, and plain-language reports can be used to ensure that the findings are contextualized and culturally appropriate.

Examining the co-benefits to both mental health and community resilience is a key concept of importance in strengths-based work at the intersection of this work. This paper contributes to a nascent field of work focusing not only on the mental health impacts of climate change on children/youth, but also seeking to build a new arena of co-benefits research on the power of taking action to build community and mental health resilience during the climate crisis.

## 6. Conclusions

A notable shift is currently underway: children/youth are increasingly engaging in risk reduction, community climate adaptation, and mitigation initiatives, and policy discussions [10]. Notable trends are the “Greta Effect” [58], FridaysforFuture [59], climate strikes, BirthStrike [60], and the civil lawsuit filed by children/youth against the federal government. Children/youth are also reaching out to talk with family members, teachers, and community groups about their experiences of distress, alienation and urgency [12]; however, many adults are actively seeking guidance on how to respond, indicating that many climate conversations cannot yet be had between generations [20]. Children/youth continue to grapple with their thoughts about the future. They have a unique capacity to perceive risks that are particular to their age and circumstances, and to propose child- and youth-friendly ways to overcome them [12,61]. Because of this, children/youth can offer ideas that may help to resolve challenges to local infrastructure, offer insights into children and youth’s behaviors and resources to help strengthen community early warning systems, increase climate change awareness amongst their peer groups and with their families, and even model how to take doable, local actions on climate change that make a notable difference [62]. When governments, health care services, and communities enable and support their participation, children/youth can and do meaningfully contribute [63]. Climate change is irrevocably transforming their world; therefore, supporting the agency of children/youth to participate in developing creative solutions and resilient responses is appropriate and timely [4].

This research shows that when the mental health and wellbeing of children and youth is considered, from their sense of distress about an unknown future through to developing strategies for adapting to, and coping with, a chaotic climactic future, communities are given guidance on how to build whole and healthy futures. This paper has explored factors such as engaging children/youth as partners, researchers, and knowledge producers at a time when they are calling for the adult world to take urgent action to ensure that they will grow up and learn how to sustainably steward life on a healthy planet. This paper has also acknowledged that children/youth are experts in their own lives, and if included in the means of knowledge production, community-based decision making, policy development, and program delivery they will offer fresh perspectives to old challenges and learn to be change makers in their own right. The hope is that this work will foster a deepening understanding around children and youth’s responses to climate change and stave off despair through facilitating active efforts to build transformative responses to climate change. Through this integrated approach, taking action on climate change can not only improve children/youth’s health and wellbeing but also help to build and sustain healthy communities. As such, considering the Social and Ecological Determinants of Health when looking at both how climate change is impacting children and youth’s mental health and how to better support youth and children in the future are complementary practices.

## Supplementary Materials

The following are available online at https://www.mdpi.com/article/10.3390/ijerph18094573/s1, Table S1: The Interplay between Social and Ecological Determinants of Mental Health for Children and Youth in the Climate Crisis.
