# Supplementary material for: The Interplay between Social and Ecological Determinants of Mental Health for Children and Youth in the Climate Crisis
Source: Int J Environ Res Public Health. 2021 Apr 26;18(9):4573. doi: 10.3390/ijerph18094573 (PMC8123462; doi:10.3390/ijerph18094573)
Supplement: Supplementary file 1 [file ijerph-18-04573-s001.zip › ijerph-1183418-supplementary.pdf]

Table S1. The Interplay between Social and Ecological Determinants of Mental Health for Children and Youth in the Age of Climate Crisis.

Theme/Research Question	Title	Authors	Population (including size)	Country	Study Design	Result as it relates to the theme
1. Direct and indirect impacts of climate change on children/youth's mental health in Canada	A Review of the Consequences of Global Climate Change on Human Health	Ki-Hyun Kim, Ehsanul Kabir & Shamin Ara Jahan	Everyone, particular attention to children, elderly, communities in poverty	Global	Review	"The impact on mental health induced by extreme weather conditions (like hurricanes, tornados, floods, fires, drought, tsunamis, etc.) is expected to be reflected by anxiety, post-traumatic stress, depression, etc." pp.11)
	Report of the intergovernmental panel on climate change: implications for the mental health policy of children and adolescents in Europe—a scoping review*	Clemens, V., von Hirschhausen, E., & Fegert, J	Children and adolescents	Global - European focus	Scoping Review	Review of direct and indirect impacts of climate change on children and youth concluding that focus should include mental health support.
	Affective Pedagogies: Foregrounding Emotion in Climate Change Education*	Audrey Bryan	Learners	Global	Commentary	"...new emotions, including 'ecological grief' and heightened levels of climate-related anxiety amongst young people have been reported…"

	Children and youth in the era of climate change*	Kiaras Gharabaghi & Ben Anderson-Nathe	Children and youth	Global	Editorial	Children and youth are disproportionately affected by climate change but have less agency to take action, as such, researchers and adults have a duty to address climate change.
	Climate Change and Children's Health*	Aaron Bernstein & Samuel Myers	Children	Global	Review	Children's health in both developing and developed countries is negatively affected by climate change events which impact food sources, quality of air and water, and disease.
	Climate Change and Children's Health: A Commentary*	Fiona Stanley & Brad Farrant	Children and young people	Global	Commentary	Climate change is a wicked problem driven by various inequalities which affects the physical and mental health of children around the world.
	Climate change and Mental Health*	Janna Trombley, Stephanie Chalupka & Laura Anderko	Various equity deserving populations (e.g., elderly, children, women, those living in poverty)	US	Review	Climate change results in various mental health impacts including anxiety, stress, depression, violence, and impacts on community identity.
	Climate change is a major stressor causing poor pregnancy outcomes and child development	David Olson & Gerlinde Metz	Children and pregnant women	Global	Review	Climate change directly and indirectly affects mental health in cumulative ways.

	Climate Change, Climate Justice, and Children's Mental Health: A Generation at Risk?*	Stephanie Chalupka, Laura Anderko, & Emma Pennea	Children	Global	Commentary	Climate change disproportionately impacts children in various direct and indirect ways, but not all children are equally impacted, as those living in poverty are more impacted than others.
	Climate Change, Conflict, and Children	Richard Akresh	Children	Global	Commentary	Climate change leads to increased violence and conflict, which disproportionately impacts children in various life stages.
	Extreme weather-driven disasters and children's health	Daniel Martinez Garcia & Mary Sheehan	Children <18 years of age	Global	Narrative literature review and conceptual model creation	Despite experiencing various detrimental impacts of climate change, children continue to show extraordinary resilience
	Impacts of Natural Disasters on Children	Carolyn Kousky	Children	Global	Review	A climate event's impact on children's mental health relies on various social and economic factors.
	Mapping vulnerability to climate change-related hazards: children at risk in a US–Mexico border metropolis	Timothy Collins et al.	Children	US-Mexico region; El Paso County	Mapping Analysis	The impacts of climate change on children will continue to increase over future decades.

	Mental health impact for adolescents living with prolonged drought*	John Dean & Helen Stain	Adolescents (age 11-17)	New South Wales	Mixed methods using focus groups and questionnaire	Climate change leads to feelings of grief, loss, and various mental health concerns among adolescents
	The Psychological Effects of Climate Change on Children*	Susie Burke, Ann Sanson, & Judith Van Hoorn	Children	Global	Review	The direct and indirect effects of climate change on mental health can impact their mental health functioning in adulthood.
	Will boys' mental health fare worse under a hotter climate in Australia?	Ying Xu, Sarah Wheeler, & Alec Zuo	Children (age 6-11)	Australia	Literature review and structural equation modelling; mediation analysis	Direct and indirect effects of climate change worsen children's mental health. The impact on boys' mental health is mostly related to hyperactivity and peer problems. The impact on girls' mental health is influenced by family and socio-economic factors.
	Healthy Air, Healthy Brains: Advancing Air Pollution Policy to Protect Children's Health	Devon Payne-Sturges et al.	Children	US	Commentary	Children are affected prenatally and throughout childhood to climate change.

	The International Society for Children's Health and the Environment Commits to Reduce Its Carbon Footprint to Safeguard Children's Health.	Eskenazi, B., Etzel, R., Sripada, K. Et al	Children	Global, with some focus on US	Brief commentary	Children are impacted by climate events both physically and mentally. A call to action for policy to include factors that affect future generations and to take a multisectoral approach.
2. Children/youth's perceptions or views of climate change and how this impacts their mental health and wellbeing	Children's Perceived Water Futures in the United States Southwest	Vins, H., Wutich, A., Brewis, A., Beresford, M., Ruth, A., & Roberts, C.	Teachers (n=74) And students (n=1560)	US	Qualitative - visual content analysis	Analyzed schoolchildren's perceptions of the future of water through arts-based methods and differences of perception
	Climate change-the thief of childhood*	Grauer, S	Students, children and youth	US	Commentary	Children experience eco-anxiety about the climate crises and need to be empowered to share their own voice and take action.
	"Cycling – exercise or trying to stop pollution": methods to explore children's agency in	Chadborn, N., Gavin, N., Springett, J., & Robinson, J.	Children (age 10-11 years) (n=67)	UK	Qualitative - drawing methods	Explored perceptions and awareness of climate change for children using photography and drawing methods.

	health and climate change					
	Educating for resilience: parent and teacher perceptions of children's emotional needs in response to climate change*	Baker, C., Clayton, S., Bragg, E.	Parents, teachers, and children (n=141)	Australia	Mixed methods - online survey with analysis	Explored emotions around climate change for children and discovered barriers to speaking with children about climate change
	In their own words: Young people's mental health in drought-affected rural and remote NSW	Carnie, T., Berry, S., Blinkhorn, S., & Hart, C.	Young people, parents, and service providers	Australia	Qualitative - content analysis	Reported youths mental health and overwhelm impacted by drought
	Regulating worry, promoting hope: How do children, adolescents, and young adults cope with*	Ojala, M.	Youth in early, middle, and late adolescence (~11-23 years old) (n=348)	Sweden	Mixed methods - questionnaire	Explored youths' perception of climate change (i.e.: thoughts about hope, trust, severity) to understand coping strategies.

	Climate-sensitive health priorities in Nunatsiavut, Canada*	Harper, S., Edge, V., Ford, J., Willox, A., Wood., M., IHACC Research Team., & McEwen, S.	Youth (n=13), adults (n=128), elders (n=63)	Canada	Mixed methods - Photovoice, in-depth interviews, and surveys	Used photovoice to understand relationships between social determinants of health and climate with an eco-health perspective.
	"From this place and of this place:" Climate change, sense of place, and health in Nunatsiavut, Canada*	Cunsolo Willox, A., Harper, S., Ford, J., Landman, K., Houle, K., Edge, K., Rigolet & Inuit Community Government	Community members	Canada	Qualitative - In depth interviews (n=72) Questionnaire (n=112)	A multi-year community case study explored the impacts of climate change on health. Emphasizing climate-health planning specific to regional contexts.
	Images of the child and environmental risk: Australian news photography of children and natural disasters, 2010–	Butler, R	Children	Australia	Commentary	Uses photographs to explore the impact of children in natural disasters, revealing many anxieties and close relationships to the natural world.

	2011					
	Indigenous Values and Health Systems Stewardship in Circumpolar Countries*	Chatwood, S., Paulette, F., Baker, R., et al	Community experts (n=10)	Norway, Finland, United States and Canada	Mixed methods	Explored values of Indigenous peoples and identified 9 themes: humanity, community voice, empowerment, respect, cultural responsiveness, teaching, nourishment, kinship and holism.
	Learning about climate change in, with and through art*	Bentz, J.	High school students (age 16-18) (n=70)	Portugal	Arts-based methods	Using arts communication as a teaching tool for climate change education and understanding with students.
	Protective factors for mental health and well-being in a changing climate: Perspectives from Inuit youth in Nunatsiavut, Labrador*	MacDonald, J., Cunsolo Willox, A., Ford, J. et al	Youth (aged 15-25)	Canada	Qualitative - interviews	Explored factors which improved and fostered resilience in youth and application for adaptive response to climate change.
	The impact of climate change on youth depression and mental health*	Majeed, H., Lee, J.	Youth (generally)	Canada	Commentary	Raises awareness about youth depression and anxiety being exacerbated by events and situations caused by the climate crisis.

	The Psychometric Equivalence of the Personal Wellbeing Index School-Children for Indigenous and Non-Indigenous Australian Adolescents (3)	Tomyn, A., Fuller Tyszkiewicz, M., & Norrish, J	Adolescent (age 12-19) Indigenous (n=1378) non-Indigeneous (n=6,401) 'non-Indigenous Mainstream' (n=983)	Australia	Mixed methods	Offers a measurement tool for wellbeing in Indigenous and non-Indigenous Australian adolescents
	Development and validation of a measure of climate change anxiety	Clayton, S., Karazsia, B.	Young adults mostly between 25-34 Also included 18-24 (n=194) and 75+ (n=3)	US	Exploratory factor analysis	Explores a tool to measure psychological impacts of climate change, and explains how climate change anxiety is present amongst younger adults.
3. Ways to take action and build resilience on climate change in children/youth	A Discussion of Critical Issues in Environmental Education: An Interview with Dianne Saxe	Karen Acton & Dianne Saxe	Youth	Global	Interview	Teachers and educational policy makers need to send youth into the future with information about the climate crisis and help them build skills to take action.

	Beyond Bushfires	Lisa Gibbs et al.	Adults, adolescents, and children age 4 and above	Australia	Study Protocol - mixed methods longitudinal study	Facilitating community and social connection is a protective factor to the effects of climate change.
	Children and natural disasters	Atle Dyregrov, William Yule, & Miranda Olff	Children	Global	Editorial	Parents need to be involved in teaching children about climate change disasters and how to cope through those events.
	Children, reproductive labor, and intergeneration al solidarity	Kate Cairns	Children		Comment on Newberry and Rosen	
	Climate Change and Children: Health Risks of Abatement Inaction, Health Gains from Action*	Anthony McMichael	Children	Global	Review	To mitigate the negative physical and mental impacts of climate change on children, the education system must improve children's ecological understanding.
	Climate Change and Children's Health—A Call for Research on What Works to	Zhiwei Xu et al.	Children (under 15 years of age)	Global	Review	Effective climate change educational programs, post-disaster counselling, and incorporation of pediatrician advocacy are important to protecting the health of children experiencing climate events.

	Protect Children*					
	Climate Change Challenges and Opportunities for Global Health	Jonathan Patz et al.	All populations (including children)	US	Review	Given that children face disproportionate risks of climate change consequences, protective strategies need to be taken including supporting social networks, and providing adequate mental health support post-disaster.
	Climate Change: Implications for Parents and Parenting*	Ann Sanson, Susie Burke, & Judith Van Hoorn	Children and youth	Global	Tutorial	The mental health response and resiliency of youth and children experiencing climate change events is largely determined by how parents and guardians respond to such events.
	Collaborative approaches to wellness and health equity in the Circumpolar North: Introduction to the Special Issue*	Nathaniel Pollock & Ashlee Cunsolo	Indigenous peoples, including Indigenous youth	Circumpola r North	Editorial	"importance of ensuring that Inuit youth are supported to become the driving forces behind programs to support youth resilience, and that youth creativity and passion is essential to generating knowledge and create a collective vision for a better future" (pp.3)
	Hands-On Ecological	Gary Nabhan et	All populations	Global	Review	Young people benefit from green prescriptions and engagement in reciprocal

	Restoration as a Nature-Based Health Intervention: Reciprocal Restoration for People and Ecosystems	al.	including young people			restoration of land health and human health.
	Health adaptation policy for climate vulnerable groups: a 'critical computational linguistics' analysis	Seidel, B. & Bell, E.				
	Impact of awareness and concerns of climate change on children's mental health: a scoping review protocol	Gina Martin, Kristen Reilly, & Jason Gilliland	Children (3-19 years of age)	Global	Review	Children are engaging in climate change advocacy globally. "An increased awareness and concern represents a threat to the mental health and wellbeing of children, but on the other hand, it can trigger pro-environmental behavior for children and their families" (pp.517).
	Improving the mental health of rural New South Wales	Craig Hart, Helen Berry, & Anne	Aboriginal communities, older farmers, young people,	Rural New South Wales	Community engagement	To protect the mental health of children experiencing climate change events, there news to be early identification and referral to mental health services and inclusion of

	communities facing drought and other adversities	Tonna	women			climate event in school curriculum
	Intersectoral approaches and integrated services in achieving the right to health for refugees upon resettlement: a scoping review protocol	Javadi, D., Langlois, E.,<br>Ho, S., et al			Scoping review	
	Our House Is on Fire: Child and Adolescent Psychiatrists in the Era of the Climate Crisis*	Pinsky, E., Guerrero, A., & Livingston, R.	Children and adolescents	Global	Commentary	Child and adolescent psychiatrists have a role to play in encouraging and supporting children and youth to be resilient in situations related to climate change impacts - direct and indirect.
	Raising Children to Cope with Climate Change?*	Eichler , M.	Children	Canada	Commentary	Explores coping strategies and important factors for children's wellbeing and raise for resilience and enjoyment.
	Responding to the Impacts of the Climate Crisis on	Sanson, A., Van Hoorn, J., &<br>Burke, S.	General, focus on children, youth, and students.	Global	Commentary	Children and students have historically not played a large role in the climate crisis conversation, yet climate strikes have revealed young people's perceptions,

	Children and Youth*					anxieties, and organized activism. Call to action for child health professionals to support children and understand the complexities.
	Rights, justice, and equity: a global agenda for child health and wellbeing	Goldhagen, J., Shenoda, S., Oberg, C., el al.	General, focus on children	Global	Viewpoint	“Child rights-based approaches will be required to enhance the response to the civil-political, social, economic, and cultural determinants of these global child health issues.” (pp. 1)
	The Climate Change and Health Adaptation Program: Indigenous climate leaders’ championing adaptation efforts*	Richards, G., Frehs, J., Myers, , E., & Van Bibber , M.	Indigenous communities	Canada	Commentary	“This paper demonstrates three examples of community based projects to mitigate and adapt to the health impacts of climate change to demonstrate climate change resiliency within Indigenous communities.” (pp.1)
	The impact of climate change and natural disasters on vulnerable populations: A systematic review of literature*	Benevolenza, M., DeRigne, L.	Variety of demographic groups, including children	US	Systematic Review	Children are included in ‘vulnerable’ populations that can experience impacts of climate change both physically and psychologically. A call to action for politicians and health professionals work to improve outcomes for vulnerable groups.

	Youth Engagement in Climate Change Action: Case Study on Indigenous Youth at COP24*	MacKay, M., Parlee, B., & Karsgaard, C.	Youth (n=14)	Canada	Qualitative-interviews	Gathered insights on Indigenous youth engagement at the United Nations Conference of Parties (COP24) and the importance of community and social supports to encourage youth leaders and climate activism
	A systematic review of climate change education: giving children and young people a 'voice' and a 'hand' in redressing climate change*	Rousell, D., & Cutter-Mackenzie-Knowles, A.,	Children and young people	Australia	Systematic Review	Calls for participatory research to engage young people on climate change in ways that are relevant socially, culturally, politically, and geographically.
	Climate anxiety in young people: a call to action*	Wu, J., Snell, G., & Samji, H.	General population, focus on young people	Canada	Commentary	Health professionals and politicians have a role to play to understand and support young people as agents of change
	Climate science curricula in Canadian secondary schools focus on human warming, not	Wynes, S., & Nicholas, K.	Secondary school students (Grade 9-12)	Canada	Curriculum analysis	Reviewed school curriculums across all provinces to reveal trends and extent of climate change education.

	scientific consensus, impacts or solutions*					
	Focus groups with young people: a participatory approach to research planning*	Bagnoli, A., & Clark, A	Youth (age 13)	UK	Participatory action research, longitudinal	Explored practices on engaging youth in participatory action research in a longitudinal study
	Imagining future worlds alongside young climate activists: a new framework for research*	Bowman, B.	General	UK	Commentary	Calls for a focus on methods that center youth voices in participatory action approaches, some methodologies do not represent the uniqueness of youth political participation and focus on a 'top-down' approach.
	Youth-led climate strikes: fresh opportunities and enduring challenges for youth research – commentary	Wood, B.	General	N/A	Commentary	Builds on Bowmans commentary to critique research methodologies that perpetuate a deficit-based approach or "cynicism", including how one reports on youth's actions towards climate change. A need to understand the community as a whole, as opposed to isolated individuals.

	to Bowman					
	Children's protest in relation to the climate emergency: A qualitative study on a new form of resistance promoting political and social change*	Holmberg, A., Alvinius, A.	Greta Thunberg and children/youth	Global, with some Sweden focus	Qualitative - Thematic analysis	Explores children expressing political autonomy in reaction to climate change. "Two themes, new to the literature, are identified: (1) need for political and social change focusing on the climate emergency, resistance towards laissez-faire behaviour and exhortations, and (2) resistance targets including the political leaders, capitalist ideologies and older generations."
